# Association between vascular aging and cardiovascular-kidney-metabolic syndrome

**DOI:** 10.3389/fendo.2025.1665836

**Published:** 2025-11-26

**Authors:** Shuyi Zeng, Henghua Cui, Wang Liao, Shiyu Qiu, Zhengdong Wang, Zijia Wu, Ming Liu, Lei Chen

**Affiliations:** Department of Cardiology, The First People’s Hospital of Yulin, the Sixth Affiliated Hospital of Guangxi Medical University, Yulin, China

**Keywords:** cardiovascular–kidney–metabolic syndrome, cardiorenal syndromes, metabolic syndrome, vascular stiffness, pulse wave velocity

## Abstract

**Background:**

The concept of Cardiovascular-Kidney-Metabolic syndrome (CKM), an intricate interplay among metabolic risk factors, chronic kidney disease (CKD), and the cardiovascular system, was introduced by the American Heart Association (AHA). This indicates a markedly increased susceptibility to various organ dysfunctions and adverse cardiovascular events. In CKM management, reliable markers are critical. The purpose of this study was to investigate the relationship between ePWV and mortality and prognosis in patients with CKM.

**Methods:**

This study included 14,372 participants from the National Health and Nutrition Examination Survey (NHANES) from 2005 to 2018. ePWV assessed vascular aging and arterial stiffness. The association between ePWV and CKM, as well as their risk of death, was analyzed using weighted logistic regression and Cox proportional hazards models. Bootstrap sampling was used to analyze the mediating effect of the Triglyceride-Glucose Index (TyG) between ePWV and CKM. The Stage 4 ePWV threshold was further validated using an independent retrospective cohort of 3,157 patients from the cardiology department of a tertiary hospital in southern China.

**Results:**

The RCS analysis demonstrated a nonlinear positive association between estimated pulse wave velocity (ePWV) and cardiovascular–kidney–metabolic syndrome (CKM) (P < 0.01). Subgroup analysis indicated that this association was strongest among young adults aged 20–40 years (P < 0.01), whereas no significant association was observed in elderly individuals over 75 years old (P = 0.791). Kaplan–Meier curves showed that individuals with higher ePWV had significantly worse survival than those with lower ePWV. Further mediation analysis revealed that the triglyceride–glucose (TyG) index mediated 25.23% of the association between ePWV and CKM. In the external validation cohort, the ePWV threshold corresponding to CKM Stage 4 was 8.52, which was consistent in direction and statistical significance with the NHANES-derived model.

**Conclusion:**

Elevated ePWV is nonlinearly associated with increased CKM risk, particularly among young adults. This finding provides insights into managing arterial stiffness and preventing CKM progression in individuals at high risk of early cardiovascular disease.

## 1 Background

The components of CKM syndrome are likely to be interconnected through common pathophysiological pathways. Metabolic risk factors, including obesity and diabetes, are tightly linked to chronic kidney disease (CKD) and impair cardiovascular function. According to the American Heart Association (AHA) Presidential Advisory issued in October 2023, CKM syndrome is defined as a systemic condition characterized by the pathophysiological interactions among metabolic risk factors, CKD, and the cardiovascular system (1). These interactions contribute to a high incidence of multiorgan dysfunction and adverse cardiovascular outcomes. Unhealthy lifestyle behaviors and poor self-management further increase the risk of CKM syndrome and its adverse consequences. To improve monitoring of CKM-related disease progression and mortality, it is essential to identify prognostic parameters with potential value beyond traditional assessment indicators.

Arterial stiffness (AS) is a major determinant of cardiovascular morbidity and mortality in patients with hypertension. Several risk factors, including aging, hypertension, vascular calcification, inflammation, and diabetes, contribute to its development. Arterial stiffness increases with age and is associated with a higher risk of multiple adverse outcomes, such as heart disease, dementia, and kidney disease (2, 3). While arterial stiffness associated with aging is well known, a number of mechanisms have been identified that contribute to the development of arterial stiffness (4). The deposition of hormones/regulatory peptides and their receptors, pro-inflammatory cytokines/chemokines, and calcium in the vascular system have all been identified as potential mechanisms of arterial stiffness (5–7). Pulse wave velocity (PWV) is considered to be the gold standard indicator for the assessment of arterial stiffness. The estimated pulse wave velocity (ePWV) is a calculated index derived from empirical equations incorporating age and blood pressure, designed to approximate carotid–femoral pulse wave velocity and reflect arterial stiffness. It is observably marked as a criterion with significant standing in determining vascular rigidity (8). Recently, ePWV has emerged as an alternative to directly measured PWV, providing information beyond traditional cardiovascular risk predictors and offering potential for early prevention and prognostic evaluation of cardiovascular events (9).

However, the associations among arterial stiffness, vascular aging, and the prevalence and clinical outcomes of CKM remain poorly understood. The present study aimed to investigate the potential utility of ePWV, an index of arterial stiffness, in characterizing CKM and predicting its outcomes.

## 2 Methods

### 2.1 Data source and study population

The National Health and Nutrition Examination Survey (NHANES) employs a dual methodology that combines structured interviews and physical examinations to evaluate the health and nutritional status of adults and children in the United States. The interviews collect demographic, socioeconomic, dietary, and health-related information, while the examinations provide objective clinical and laboratory data. The NHANES protocol is approved by the National Centre for Health Statistics (NCHS) Ethics Review Board, and all participants provide written informed consent. The survey is conducted in two-year cycles, and this study utilized data from seven consecutive cycles between 2005 and 2018, with mortality follow-up through December 2019. Further information about NHANES is available at https://wwwn.cdc.gov/Nchs/Nhanes/.

**Figure 1:**
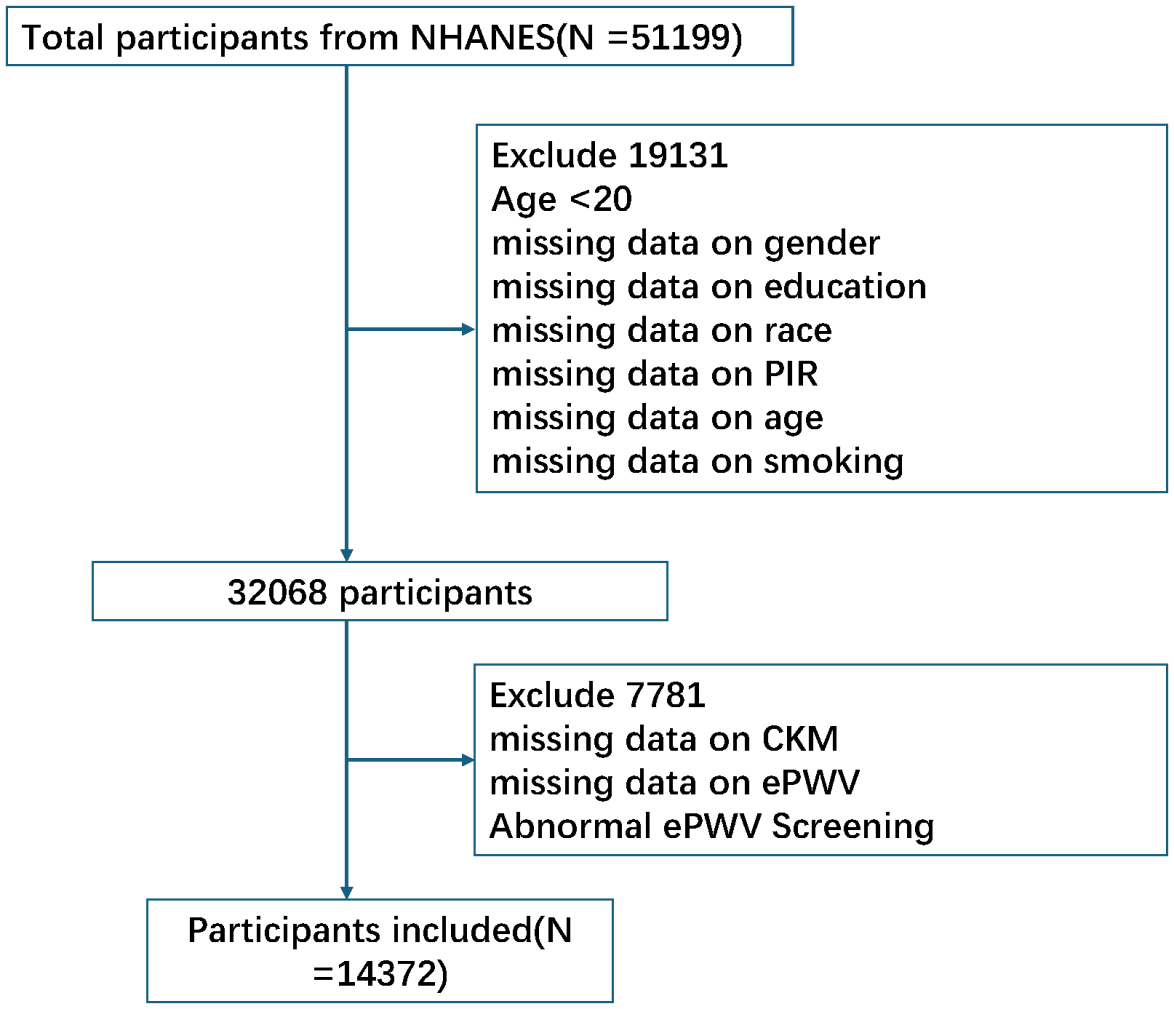
Flowchart of participant inclusion and exclusion from the NHANES 2005–2018 dataset.

Initially, 51,199 participants from NHANES were considered. After excluding individuals younger than 20 years or those with missing CKM-related diagnostic data, incomplete ePWV information, insufficient mortality follow-up, or uncertain medical history, 14,372 participants were included in the final analysis. Uncertain medical history referred to incomplete, inconsistent, or unreliable information regarding CKM-related conditions—including cardiovascular disease, diabetes, chronic kidney disease, obesity, and hypertension—or missing/conflicting laboratory and examination data required for CKM classification. These participants were excluded to ensure diagnostic accuracy and analytic reliability. Missing data were evaluated for all covariates, and when the proportion of missing values was below 15%, multiple imputation by chained equations (MICE) was applied to preserve the largest possible sample size and minimize bias from incomplete data. The participant selection process is illustrated in **Figure 1**.

### 2.2 Measurement of ePWV

ePWV, a non-invasive measure derived from age and mean blood pressure, was used to assess arterial stiffness, an indicator of vascular ageing (9, 10). The ePWV was calculated from age and mean blood pressure (MBP) using the following equation: 9.587 − 0.402 × age + 4.560 × 10^-^³ × age² − 2.621 × 10^-5^ × age² × MBP + 3.176 × 10 ³ × age × MBP − 1.832 × 10^-^² × MBP. MBP was calculated as diastolic blood pressure + 0.4 × (systolic blood pressure − diastolic blood pressure).

### 2.3 Outcome definition

CKM was assessed using the American Heart Association (AHA) Presidential Advisory(1). Each CKM stage (0–4) was operationalized using National Health and Nutrition Examination Survey (NHANES) variables, following the AHA criteria that integrate metabolic risk factors, kidney function, and cardiovascular status. The detailed mapping of CKM stages, including variable definitions, diagnostic thresholds, and decision rules, is provided in **Supplementary Table S1**. These definitions were used to determine the outcome variable in all subsequent analyses. For missing data, we employed a complete-case analysis approach, excluding participants with missing key variables for CKM staging (e.g., eGFR, albuminuria, or CVD history). No imputation was performed, and “unknown” responses were treated as missing.

### 2.4 Ascertainment of mortality and follow-up

Identification of mortality status was achieved through the synchronization of NHANES datasets and records maintained by the National Death Index (NDI), accessible via the portal at https://www.cdc.gov/nchs/data-linkage/mortality-public.htm. From the NDI’s repository, participants’ survival or demise classification was ascertained accordingly. The International Classification of Diseases, Tenth Revision (ICD-10), was utilized to define the underlying causes of death. Cardiovascular mortality was classified explicitly by the NCHS as deaths attributed to heart disease, identified by ICD-10 codes I00-I09, I11, I13, and I20-I51 (11).

### 2.5 External validation cohort

An external validation cohort was constructed using data from a retrospective study conducted in the Department of Cardiology at a tertiary hospital in southern China. A total of 3,157 patients were included after excluding individuals with missing information on CKM staging or ePWV. The cohort primarily consisted of middle-aged and older adults (mean age 47.98 ± 12.88 years), with 57.43% male participants. The prevalence of hypertension, diabetes, and chronic kidney disease was 45.62%, 28.83%, and 15.78%, respectively. CKM classification was defined using the same diagnostic criteria as applied in the NHANES cohort to ensure consistency. This independent dataset was used to validate the ePWV cutoff derived for Stage 4 CKM patients in the NHANES population. The same modelling framework and covariate adjustment strategy were adopted to ensure comparability between the two cohorts. For ethical considerations, all NHANES data are publicly available and de-identified, with informed consent obtained by the National Centre for Health Statistics (NCHS) at the time of data collection; no additional ethical approval was required for secondary analyses. The external validation cohort, derived from anonymized hospital records, was approved by the Institutional Review Board of The First People’s Hospital of Yulin (Approval No. YLSY-IRB-SR-2025114), which formally waived the requirement for individual informed consent in accordance with national and institutional regulations.

### 2.6 Covariates

Demographic information, including age, sex, race, educational attainment, marital status, family poverty income ratio (PIR), body mass index (BMI), and tobacco and alcohol use, was collected using standardized questionnaires and a computer-assisted interview system. See the NHANES Laboratory/Medical Technologists Procedures Manual for more details (12). Diabetes mellitus (DM) encompasses either an individual’s self-disclosure of the condition, their reporting of antidiabetic medication consumption, or a fasting plasma glucose concentration of≥ 126 mg/dL. Cardiovascular diseases (CVD) in discourse include heart failure, coronary artery disease, episodes of angina pectoris, myocardial infarctions, and cerebrovascular accidents or strokes within their scope. According to the guidelines set by the Kidney Disease: Improving Global Outcomes (KDIGO), estimates of populations affected by chronic kidney diseases were undertaken. Hyperlipidemia, when defined within these parameters, targets individuals taking lipid-reducing pharmaceuticals or those registering LDL levels at or above 140 mg/dL; this consideration also extends to males exhibiting HDL < 40 mg/dL and females whose HDL < 50 mg/dL. Regarding hypertension, its delineation involves a combination of patient-asserted medical diagnoses, the declared use of antihypertensive drugs, and recordings of systolic pressure meeting or exceeding 140 mmHg and/or diastolic pressure ≥ 90 mmHg (12).

### 2.7 Statistical analysis

Baseline information was expressed as weighted means (standard error) for continuous variables and weighted percentages for categorical variables. Intergroup differences were compared using weighted variance and chi-square tests. The relationships between ePWV and CKM were analyzed using weighted multivariable logistic regression, with covariates progressively incorporated to minimize confounding. The quartile-based grouping enables a nonparametric assessment of the dose–response relationship between arterial stiffness and CKM risk, thereby avoiding the assumption of linearity inherent in continuous models. The present study utilized univariable and multivariable weighted Cox proportional hazards models to assess the relationships between ePWV and all-cause and CKM mortality in patients with CKM. The outcomes of this analysis included hazard ratios (HRs) and 95% confidence intervals (CIs). Restricted cubic spline (RCS) regression was utilized to examine the potential for a dose-response relationship between ePWV and CKM, with the nonlinearity of the relationship being assessed via likelihood ratio tests. The investigation involved stratified analyses, which were undertaken to examine the consistency of the relationship between ePWV and CKM across diverse demographic subgroups. Subgroups were analyzed in order to explore the association between ePWV and CVD prevalence. This analysis was based on various demographic characteristics. The age of subjects, smoking status, gender, and CKM syndrome stage were considered. The effects of metabolic indicators were analyzed to see how they mediate the effects of age, smoking, and CKM. The present study, therefore, sought to evaluate the mediating effect of the Triglyceride-Glucose Index (TyG) on the relationships of age and smoking with CKM. The mediating effect was quantified by deriving a percentage, calculated as the indirect effect divided by the total effect. The hypothesis concerning the significance of the mediating effect was tested using a bootstrap sample (n = 1000) (13). All analyses accounted for the complex, multistage probability sampling design of NHANES. Sampling weights, strata, and primary sampling units (PSUs) were incorporated in accordance with NHANES analytic guidelines. Because this analysis included laboratory and examination variables collected through the Mobile Examination Centre (MEC), the MEC examination weight (WTMEC2YR) was selected based on the principle of using the smallest applicable subsample weight. When combining seven 2-year survey cycles (2005–2018), the 2-year MEC weights (WTMEC2YR) were divided by seven to construct an appropriate 14-year combined weight representing the U.S. civilian, noninstitutionalized population. Variance estimates were computed using the Taylor series linearization method, specifying SDMVSTRA as the strata variable and SDMVPSU as the primary sampling unit. All analyses were performed using the survey procedures in R (package survey) to ensure nationally representative estimates.

## 3 Results

### 3.1 Participant characteristics and inclusion/exclusion criteria

A total of 14,372 participants were included in the final analysis, representing approximately 88 million U.S. adults after weighting and nesting. Participants were stratified based on the presence or absence of CKM. Among those with CKM, 43.31% were female and 35.37% were young adults aged 20–45 years (**Table 1**). In the smoking subgroup, the prevalence of CKM was nearly twice as high as that of participants without depressive symptoms. Baseline differences between the MASLD and control groups were assessed using t-tests or chi-square tests, revealing significant differences in age, race, education level, smoking status, and other variables (P < 0.01). Additionally, ePWV scores were significantly higher in the CKM group compared to the control group (P < 0.01).

**Table 1:** Baseline characteristics of all participants were stratified by CKM.

Characteristic	Non-CKM	With CKM	P-value
ePWV	7.22 (1.63)	8.55 (2.05)	<0.001
Age			<0.001
20-45	20,754,314 (61.27%)	19,114,994 (35.37%)	
45-60	8,621,492 (25.45%)	17,053,424 (31.55%)	
60-75	3,559,941 (10.51%)	12,776,107 (23.64%)	
>75	935,461 (2.76%)	5,103,478 (9.44%)	
Gender			0.09
Female	14,669,690 (43.31%)	23,408,739 (43.31%)	
Male	19,201,518 (56.69%)	30,639,264 (56.69%)	
Race			<0.001
Mexican American	2,932,338 (8.66%)	4,344,867 (8.04%)	
Other Hispanic	1,819,709 (5.37%)	2,779,185 (5.14%)	
Non-Hispanic White	22,952,418 (67.76%)	37,663,611 (69.69%)	
Non-Hispanic Black	3,349,097 (9.89%)	5,586,402 (10.34%)	
Other Race	2,817,646 (8.32%)	3,673,938 (6.80%)	
Education			<0.001
No completion of primary school	1,422,147 (4.20%)	3,178,555 (5.88%)	
Sishu/home school/elementary school	2,982,655 (8.81%)	6,117,225 (11.32%)	
Middle school	7,201,902 (21.26%)	12,690,849 (23.48%)	
High school	10,069,751 (29.73%)	17,483,144 (32.35%)	
Above high school	12,194,754 (36.00%)	14,578,229 (26.97%)	
PIR			0.05
Under the poverty line	11,076,413 (32.70%)	18,829,787 (34.84%)	
Above the poverty line	22,794,795 (67.30%)	35,218,215 (65.16%)	
Smoking			<0.001
NO	20,387,461 (60.19%)	27,591,951 (51.05%)	
YES	13,483,747 (39.81%)	26,456,051 (48.95%)	

Data are expressed as mean ± standard error or frequency (percentage); PIR, Ratio of family income to poverty; ePWV: estimated pulse wave velocity; CKM, cardiovascular-kidney-metabolic syndrome.

### 3.2 Association of ePWV with CKM

**Figure 2:**
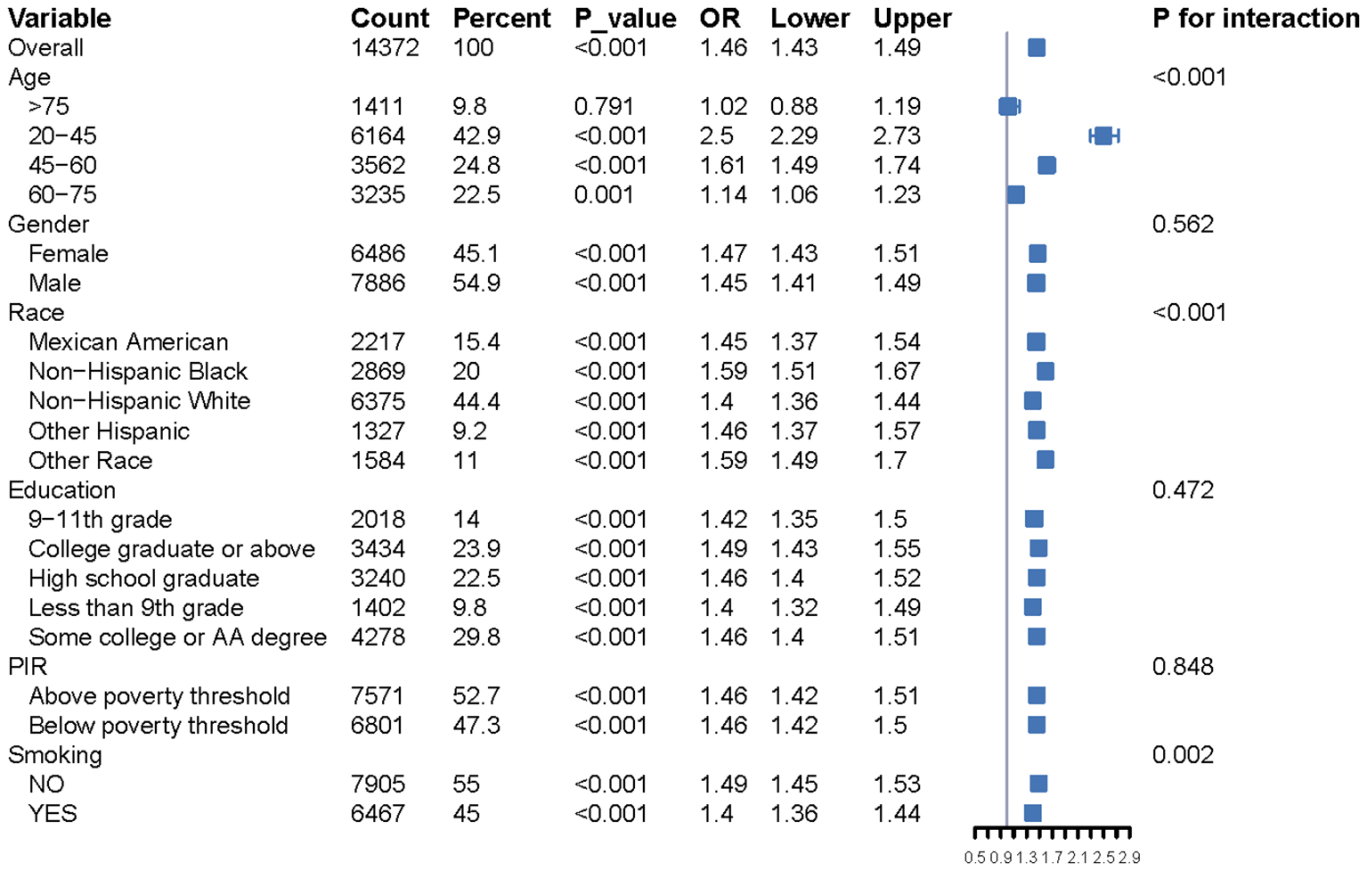
Forest plot showing univariate GLM regression analysis of estimated Pulse Wave Velocity (ePWV) and CKM prevalence across demographic and clinical subgroups.

**Figure 3:**
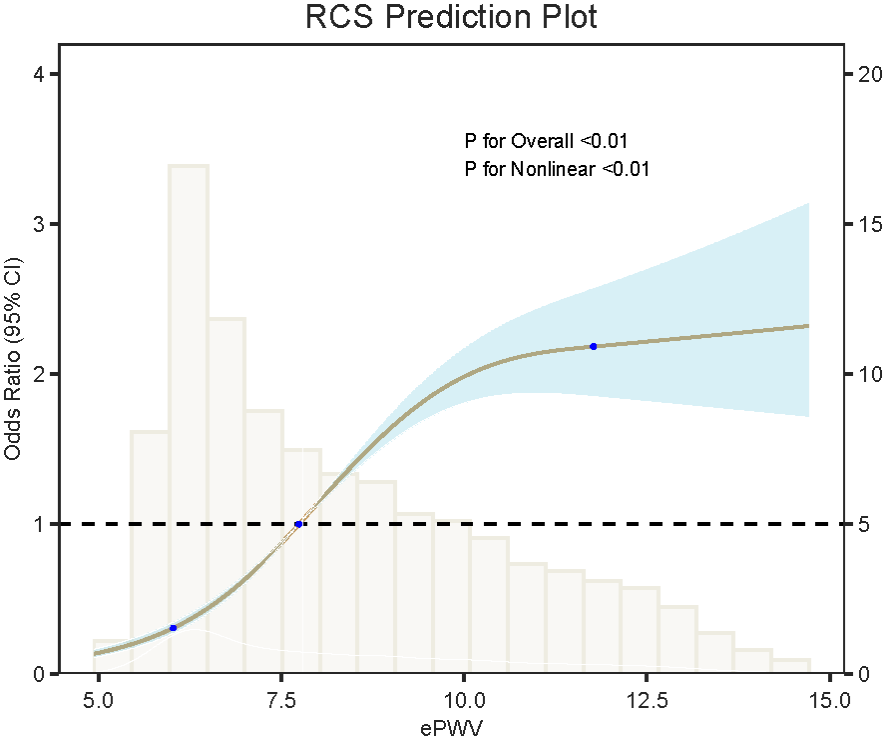
Dose–response relationship between ePWV and CKM risk using restricted cubic splines (RCS).

Subgroup analyses revealed a consistent positive association between ePWV and CKM across all subgroups, including sex, age, race, poverty-income ratio (PIR), and smoking status. Significant interactions were observed for age, race, and smoking (P for interaction < 0.01), as shown in **Figure 2**. **Table 2** summarizes the association between ePWV and CKM. After full adjustment for covariates (Model 3), the association remained significant but attenuated, with an odds ratio (OR) of 1.31 (95% CI: 1.27–1.37, P < 0.01). Participants in the highest ePWV quartile (Q4) had a substantially higher risk of CKM compared to those in the lowest quartile (Q1), with an OR of 4.68 (95% CI: 3.77–5.83, P < 0.01). This positive trend was consistent across quartiles (P for trend < 0.01). Restricted cubic spline (RCS) analysis revealed a significant nonlinear positive association between ePWV and CKM risk (P for overall < 0.01; P for nonlinearity < 0.01). As shown in **Figure 3**, CKM risk demonstrated a generally positive association with increasing ePWV values, indicating that higher arterial stiffness was related to greater cardiometabolic risk. The regression model identified an inflection point around 14.7 m/s; however, this estimate is likely influenced by the limited number of participants at the upper ePWV range and does not represent a clinically meaningful threshold.

**Table 2:** Association between the ePWV index and CKM.

	Model 1 OR (95%CI)	*P* value	Model 2 OR (95%CI)	*P* value	Model 3 OR (95%CI)	*P* value
Non-CKM	reference	–	reference	–	reference	–
CKM	1.46 (1.43-1.49)	<0.01	1.32(1.27-1.38)	<0.01	1.31 (1.27-1.37)	<0.01
Interquartile						
Quartile 1	reference	–	reference	–	reference	–
Quartile 2	2.27(2.08-2.52)	<0.01	2.06(1.56-2.29)	<0.01	2.08(1.87-2.31)	<0.01
Quartile 3	5.12(4.62-5.67)	<0.01	3.89(3.34-4.53)	<0.01	3.83(3.29-4.47)	<0.01
Quartile 4	7.68(6.88-8.57)	<0.01	4.88(3.94-6.05)	<0.01	4.68(3.77-5.83)	<0.01
*P* for trend		<0.01		<0.01		<0.01

Model 1: No covariates were adjusted; Model 2: Adjusted for gender and age; Model 3: Adjusted for age, gender, race, PIR, education, smoking; CKM, cardiovascular-kidney-metabolic syndrome.

### 3.3 Progressive association of ePWV with CKM severity

Logistic regression based on the fully adjusted model was used to assess the association between ePWV and different stages of CKM. The results demonstrated a stepwise increase in the odds ratios (ORs) with advancing CKM stages: 1.00 for Stage 1, 1.44 (95% CI: 1.41–1.47) for Stage 2, 1.61 (95% CI: 1.55–1.67) for Stage 3 (preclinical CVD), and 1.85 (95% CI: 1.79–1.91) for Stage 4 (clinical CVD), all with P < 0.01. More details can be seen in **Table 3**. Sensitivity analyses further supported this trend. The AUC values progressively increased from 0.52 in Stage 1 to 0.72, 0.84, and 0.86 in Stages 2, 3, and 4, respectively, indicating an improved discriminative ability of ePWV with increasing disease severity. Similar increasing trends were observed for sensitivity, specificity, positive predictive value (PPV), and negative predictive value (NPV), suggesting that ePWV is a robust marker across CKM stages, particularly for detecting advanced CKM with cardiovascular involvement. More details can be seen in **Table 4**.

**Table 3:** Association between estimated pulse wave velocity (ePWV) and CKM stages based on fully adjusted logistic regression models.

Results	CKM stage 1	CKM stage 2	CKM stage 3	CKM stage 4
	reference	reference	reference	reference
OR	1.00(0.96-1.03)	1.44(1.41-1.47)	1.61(1.55-1.67)	1.85(1.79-1.91)
P value	0.79	<0.01	<0.01	<0.01

CKM, cardiovascular–kidney–metabolic syndrome; ePWV, estimated pulse wave velocity. CKM stages were classified following the 2023 AHA Advisory definitions.

**Table 4:** Sensitivity analyses evaluating the discriminative performance of ePWV across CKM stages.

Models	AUC (95% CI)	Sensitivity (95% CI)	Specificity (95% CI)	PPV (95% CI)	NPV (95% CI)
CKM S1	0.52(0.51-0.54)	0.73(0.55-0.78)	0.34(0.30-0.53)	0.25(0.24-0.26)	0.81(0.79-0.83)
CKM S2	0.72(0.71-0.73)	0.72(0.68-0.76)	0.63(0.58-0.67)	0.68(0.67-0.70)	0.67(0.65-070)
CKM S3	0.84(0.82-0.85)	0.89(0.83-0.92)	0.68(0.65-0.75)	0.25(0.24-0.29)	0.98(0.97-0.99)
CKM S4	0.86(0.85-0.87)	0.89(0.80-0.91)	0.72(0.70-0.81)	0.46(0.45-0.54)	0.96(0.94-0.97)

AUC refers to the area under the receiver operating characteristic curve; CI indicates the confidence interval; PPV represents the positive predictive value; NPV denotes the negative predictive value. The optimal ePWV cut-off points were determined using the Youden index, which maximizes the sum of sensitivity and specificity.

### 3.4 Estimation and validation of ePWV thresholds by CKM stage

A retrospective study of a Southern Chinese population was conducted to construct an external validation cohort. After excluding cases with missing CKM staging and ePWV data, a total of 3,157 patients with clinical cardiovascular diseases (CVD) in CKM stage IV were included. This independent dataset was used to assess the applicability of the regression analysis results from the NHANES cohort and the estimated ePWV threshold for stage IV. Using generalized additive models (GAMs), we estimated the ePWV thresholds corresponding to different stages of CKM based on data from NHANES stages 1 to 4. After adjusting for age, gender, race/ethnicity, poverty income ratio (PIR), education, and smoking, the thresholds increased steadily with disease progression: 7.27 for Stage 1, 7.80 for Stage 2, 8.35 for Stage 3, and 8.81 for Stage 4 (all P < 0.01). To examine whether the Stage 4 threshold could be applied beyond the modeling dataset, we tested it in an independent external cohort. The threshold identified in the validation cohort was 8.52, slightly lower than the estimate from the NHANES-derived model. Despite this difference, the threshold remained statistically significant (P < 0.01), and the overall trend was consistent. More details can be seen in **Table 5**.

**Table 5:** Threshold values of ePWV across CKM stages estimated by GAM in NHANES stage 1–4 cohorts and validated in an external cohort.

CKM stage	Stage 1	Stage 2	Stage 3	Stage 4	External validation cohort
Threshold	7.27	7.80	8.35	8.81	8.52
P value	<0.01	<0.01	<0.01	<0.01	<0.01

The threshold was estimated using a generalized additive model (GAM) and adjusted for age, gender, race/ethnicity, poverty income ratio (PIR), education level, and smoking status. CKM stages were classified following the 2023 AHA Advisory definitions.

### 3.5 Kaplan–Meier and Cox regression analyses of ePWV and mortality in CKM

**Figure 4:**
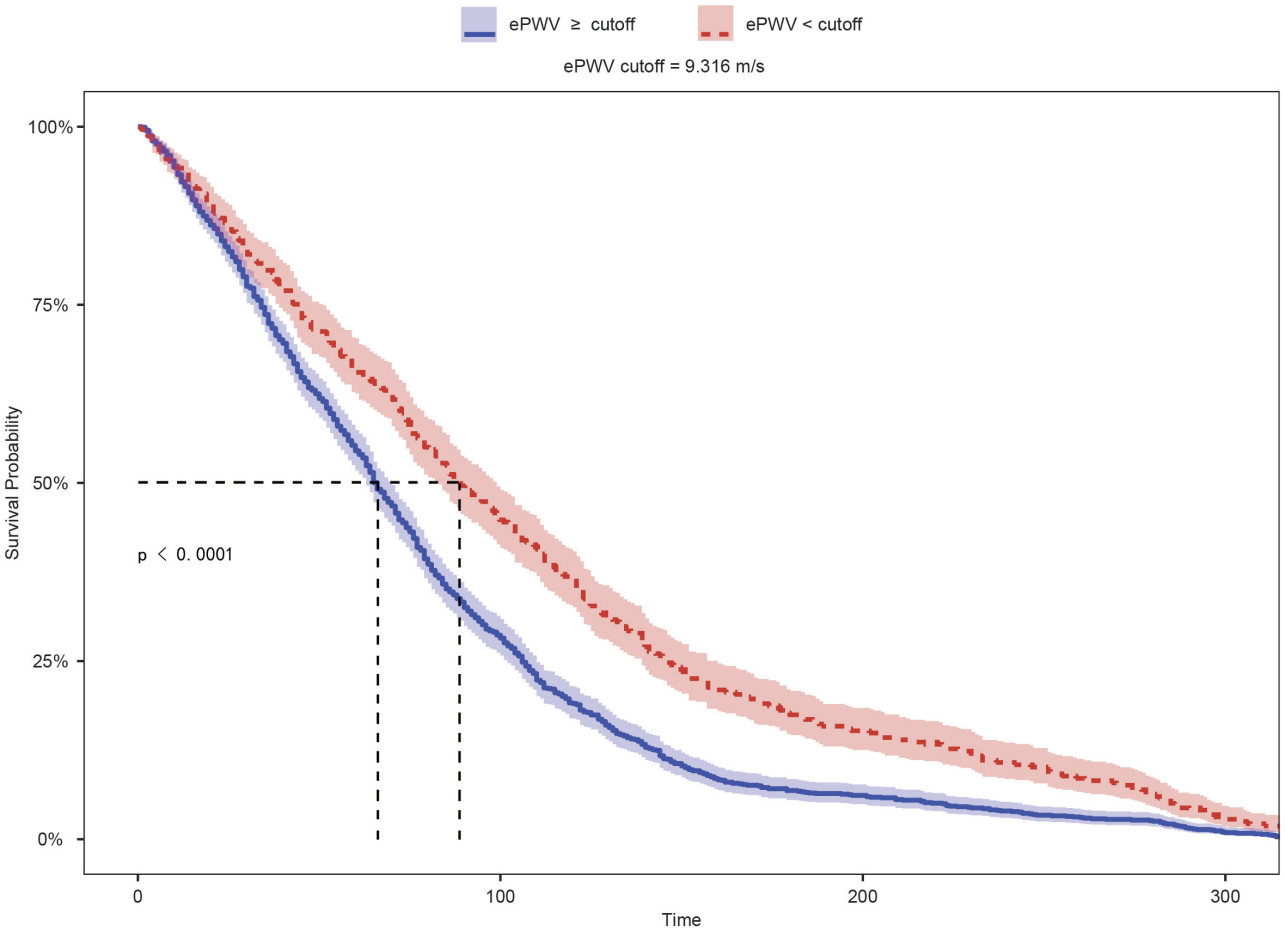
Kaplan–Meier survival curves of CKM patients stratified by ePWV level.

As shown in **Figure 4**, Kaplan–Meier survival analysis demonstrated that patients with higher ePWV levels had significantly lower survival probabilities compared with those with lower ePWV (median survival: 66 vs. 89 months; log-rank P < 0.01). The restricted mean survival time (RMST) was also shorter in the high-ePWV group (79.41 vs. 108.71 months), indicating that elevated ePWV was associated with poorer prognosis among individuals with CKM. To further examine this association, Cox proportional hazards regression models were applied to estimate the relationship between ePWV and mortality risk. As summarized in **Table 6**, higher ePWV was consistently associated with increased risks of both cardiovascular (CVD) and all-cause mortality. In the unadjusted model, each unit increase in ePWV was associated with a 43% higher risk of CVD mortality (HR = 1.43, 95% CI 1.38–1.48, P < 0.01) and a 25% higher risk of all-cause mortality (HR = 1.25, 95% CI 1.43–1.49, P < 0.01). After adjusting for demographic and clinical covariates, these associations remained robust. In the fully adjusted model, ePWV remained a significant predictor of CVD mortality (HR = 1.15, 95% CI 1.12–1.25, P < 0.01) and all-cause mortality (HR = 1.10, 95% CI 1.27–1.37, P < 0.01). A graded association was observed across ePWV quartiles, with progressively higher mortality risks in higher categories (P for trend < 0.01). Compared with participants in the lowest ePWV quartile, those in the highest quartile had a 5.91-fold higher risk of CVD mortality (95% CI 5.32–6.75) and a 3.66-fold higher risk of all-cause mortality (95% CI 2.55–3.28) in the fully adjusted model.

**Table 6:** Association between estimated pulse wave velocity (ePWV) and mortality risk in patients with cardio–kidney–metabolic syndrome. .

ePWV	Model 1 OR (95%CI)	*P* value	Model 2 OR (95%CI)	*P* value	Model 3 OR (95%CI)	*P* value
CVD mortality, HR (95% CI)	reference	–	reference	–	reference	–
Continuous	1.43 (1.38-1.48)	<0.01	1.28(1.15-1.56)	<0.01	1.15(1.12-1.25)	<0.01
Categories						
Quartile 1	reference	–	reference	–	reference	–
Quartile 2	1.28(1.11-2.52)	<0.01	1.35(1.22-2.28)	<0.01	1.56(1.36-2.58)	<0.01
Quartile 3	3.45(3.81-5.52)	<0.01	3.87(3.21-4.34)	<0.01	4.50(3.19-5.65)	<0.01
Quartile 4	6.58(5.76-7.45)	<0.01	6.25(5.88-6.45)	<0.01	5.91(5.32-6.75)	<0.01
All-cause mortality, HR (95% CI)	reference	–	reference	–	reference	–
Continuous	1.25 (1.43-1.49)	<0.01	1.18 (1.27-1.38)	<0.01	1.10 (1.27-1.37)	<0.01
Interquartile						
Quartile 1	reference	–	reference	–	reference	–
Quartile 2	1.53 (1.35-1.29)	<0.01	2.01 (1.65-2.28)	<0.01	2.54(1.99-3.04)	<0.01
Quartile 3	3.87 (3.32-5.45)	<0.01	2.93 (2.23-3.36)	<0.01	3.41(3.05-4.11)	<0.01
Quartile 4	5.39 (4.87-5.99)	<0.01	4.36 (3.83-5.01)	<0.01	3.66(2.55-3.28)	<0.01
*P* for trend		<0.01		<0.01		<0.01

ePWV, estimated pulse wave velocity; CKM, cardio–kidney–metabolic syndrome; CVD, cardiovascular disease; HR, hazard ratio; OR, odds ratio; CI, confidence interval; PIR, poverty–income ratio. Model 1: No covariates were adjusted.

Model 2: Adjusted for gender and age.

Model 3: Adjusted for age, gender, race, PIR, education, smoking.

### 3.6 TyG index partially mediates the association between ePWV and CKM

**Figure 5:**
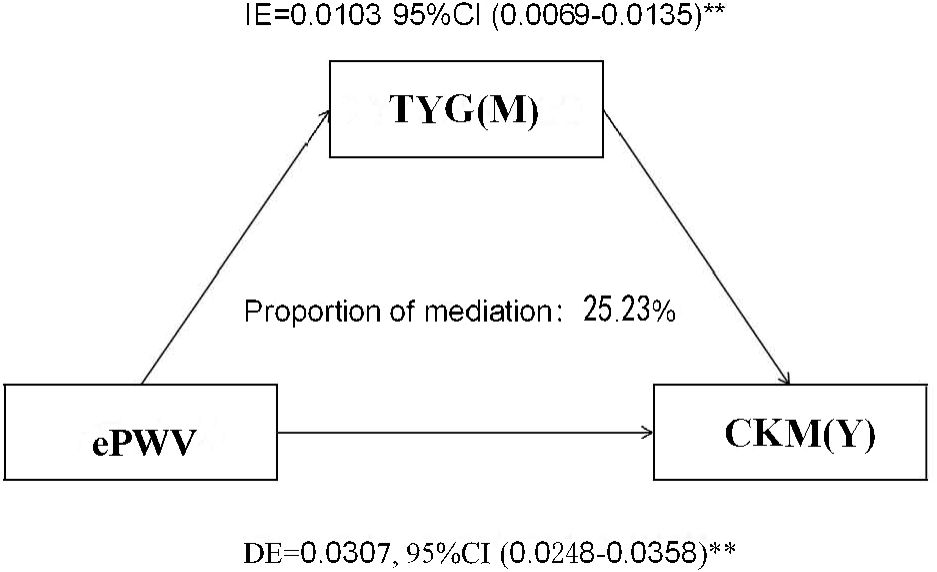
Mediation analysis of the triglyceride–glucose (TyG) index in the association between estimated pulse wave velocity (ePWV) and cardiovascular–kidney–metabolic syndrome (CKM). The TyG index significantly mediated the association, accounting for 25.23% of the total effect (P < 0.01). **statistical significance at the P < 0.01 level.

Mediation analysis was conducted to explore the potential role of the triglyceride–glucose (TyG) index in the association between estimated pulse wave velocity (ePWV) and cardiovascular–kidney–metabolic syndrome (CKM). As illustrated in **Figure 5**, TyG significantly mediated this relationship, with an indirect effect (IE) of 0.0103 (95% CI: 0.0069–0.0135, P < 0.01) and a direct effect (DE) of 0.0307 (95% CI: 0.0248–0.0358, P < 0.01). The proportion of the total effect mediated by TyG was 25.23%, indicating a substantial metabolic contribution to the observed link between arterial stiffness and CKM.

## 4 Discussion

The present study examined the complex relationship between arterial stiffness (represented by ePWV) and the prevalence and prognosis of CKM, a significant health issue with profound clinical implications. Greater arterial stiffness was associated with a higher prevalence of CKM and a corresponding tendency toward poorer outcomes. Furthermore, our study demonstrated that ePWV was independently associated with both all-cause and cardiovascular mortality in patients with CKM, even after comprehensive adjustment for demographic and clinical covariates. This finding suggests that increased arterial stiffness may play a central role in the adverse outcomes observed in CKM. The relationship between ePWV and CKM components was non-linear (p < 0.05), suggesting different risk patterns for different ePWV levels. ePWV’s effect on CKM syndrome also varies with age. This study provides substantial evidence for the role of ePWV in cardiovascular, renal, and metabolic health, thus offering novel directions for research in this field. In consideration of the established relationship between arterial stiffness, CKM, and CVD (14), it is imperative to undertake a comprehensive investigation into the association between ePWV and CVD within the context of CKM syndrome.

Age-related pathological changes in the vascular system, consequent to disease, are a pivotal factor in the elevated morbidity and mortality rates that are observed in aged populations. Age is the most significant cardiovascular risk factor in epidemiological studies, surpassing the impact of traditional risk factors (15). Vascular senescence is associated with spontaneous damage to cellular programs (e.g. senescence, inflammatory responses) and pathways (e.g. loss of protein homeostasis, impaired Nrf2-driven antioxidant responses, and DNA repair) (16). As asserted by Ndumele et al., the most prevalent etiology of CKM syndrome is the presence of excess or dysfunctional adipose tissue, with the concomitant release of proinflammatory and prooxidative products (17). The presence of ectopic fat has been demonstrated to result in the local release of active mediators, which in turn can lead to the development of compressive organ damage in the heart and kidneys. This process is associated with the induction of various health complications, including arrhythmias, myocardial and coronary artery diseases, and arterial hypertension (18, 19). The pathophysiological causes of these processes are numerous and include endothelial dysfunction, atherosclerosis, thrombosis, cardiac and renal dysfunction, and fibrosis (20, 21). All these processes favor the development of cardiovascular, cerebrovascular, peripheral arterial and renal diseases. The foundational mechanisms therein typically pertain to hemodynamic alterations, metabolic dysfunctions, inflammatory responses, and fibrotic pathways (22). Thus, inflammation-induced vascular senescence is a high-risk factor for cardiovascular events (23). In our study, a significant correlation was found between ePWV and CKM, with ePWV tending to increase as the CKM stage progressed, suggesting that vascular aging and atherosclerosis may be closely related to the progression of CKM. In early stages (e.g., CKM stages 0 and 1), ePWV is relatively low and centrally distributed, suggesting that early intervention may help slow the progression of atherosclerosis. In later stages (CKM stage 4), where its distribution is most excellent, atherosclerosis is significantly more severe and more aggressive intervention may be required. These observations align with the broader understanding that vascular aging represents not only a localized vascular process but also a marker of systemic physiological decline. Recent clinical evidence from Senguldur and Selki (2024) further supports this concept, showing that nonagenarian patients frequently exhibit overlapping cardiovascular and metabolic instability, reflecting systemic vulnerability and multi-organ dysregulation associated with advanced vascular aging (24). This reinforces the notion that increased arterial stiffness contributes to multi-system dysfunction and may underlie the coexistence of cardiovascular, renal, and metabolic abnormalities observed in CKM syndrome.

Our results demonstrate a correlation between vascular ageing and heightened arterial stiffness with elevated mortality from cardiovascular incidents within the context of CKM syndrome. Although the ePWV threshold estimated for CKM Stage 4 in the NHANES-derived model was 8.81, the external validation cohort yielded a slightly lower value of 8.52. This discrepancy may reflect population-level differences, including age structure or cardiovascular burden, and highlights the importance of independent validation. Despite this variation, the direction and statistical significance remained consistent, supporting the robustness of ePWV as a stage-specific risk marker. This finding is consistent with previous evidence that arterial stiffness parameters tend to be lower in East Asian populations for equivalent risk profiles (25). The identification of CKM patients with a vascular age greater than their actual age is therefore of particular importance for managing their prognosis. Pulse wave velocity (PWV) is a vital indicator of arterial stiffness. It is defined as an energy wave generated by cardiac contraction and propagated through the circulatory system (26). Derived through a synthesis of chronological age and mean arterial pressure, ePWV possesses notable prognostic value in assessing the gradations of arterial rigidity. When regarded as a predictor, ePWV is indicative of its potential to forecast cardiovascular incidents. Correction for the Framingham Risk Score has demonstrated that ePWV is associated with all-cause mortality, cardiovascular mortality (including fatal stroke, fatal myocardial infarction, or coronary death) and composite cardiovascular endpoints (including stroke, myocardial infarction, or coronary death) (27). Evidence from a longitudinal study of 25,066 patients without diabetes, a history of myocardial infarction (MI), stroke, heart failure or valve disease, reveals a robust correlation between ePWV and both all-cause mortality and MI, in addition to conventional risk factors (28).

In addition, we investigated the TyG-mediated relationship between ePWV and CKM and found a significant mediating effect of TyG on the relationship between ePWV and CKM occurrence. It has been shown that insulin resistance leads to disruptions in the glucose metabolic pathway, ultimately resulting in a hyperglycemic state, which in turn triggers inflammation and oxidative stress, leading to the development of atherosclerosis (29). Studies suggest that, in the early stages of atherosclerosis, insulin resistance may also contribute to the progression of atherosclerotic plaque formation by downregulating the insulin receptor–Akt1 signaling pathway, resulting in reduced activation of endothelial nitric oxide synthase (eNOS) and increased expression of vascular cell adhesion molecule-1 (VCAM-1) in arterial endothelial cells (30). Evidence has emerged indicating a correlation between insulin resistance and the promotion of smooth muscle cell proliferation. This process has the potential to result in myocardial fibrosis, consequently accelerating the onset of heart failure (31). The studies referenced above demonstrate that, notwithstanding the intricacies inherent to the pathogenesis of cardiovascular diseases, the TyG indexes of insulin resistance have the capacity to elucidate several of the underlying factors and offer a range of prospective strategies for the prevention and management of cardiovascular diseases.

The study’s primary strength lies in its large-sample cohort design, which was utilized to assess the association between ePWV and CKM initially. Secondly, the use of the NHANES database enables reliable statistical analyses due to the broad, nationally representative sample it provides, thereby ensuring the applicability of the results to wider populations. However, it is essential to acknowledge the limitations of the present study. Firstly, the present study is grounded in observational data derived from the NHANES database. It must be noted, however, that such data may be subject to selection bias, confounders, and measurement error. Second, ePWV may not fully highlight cardiovascular atherosclerosis compared with direct PWV measurements. Finally, given that this study was conducted as a single-center study, there is a possibility that unmeasured confounders may have been missed despite the implementation of multivariate adjustment and subgroup analysis.

## 5 Conclusion

Higher estimated pulse wave velocity is independently associated with a greater prevalence of CKM, suggesting that arterial stiffness and vascular aging may represent key components of CKM. These findings underscore the importance of incorporating vascular age metrics, such as ePWV, into CKM staging frameworks to strengthen risk assessment and early prevention. However, further longitudinal studies are needed to validate these associations and elucidate their causal relationships.

## Data Availability

Publicly available datasets were analyzed in this study. This data can be found here: https://www.cdc.gov/nchs/nhanes/index.html.
