# Supplementary material for: Association between vascular aging and cardiovascular-kidney-metabolic syndrome
Source: Front Endocrinol (Lausanne). 2025 Nov 26;16:1665836. doi: 10.3389/fendo.2025.1665836 (PMC12689377; doi:10.3389/fendo.2025.1665836)
Supplement: Supplementary file 1 [file Table1.docx]

**Materials and methods**

**Table S1. Definition of cardiovascular-kidney-metabolic syndrome stages**

| **CKM Stage 0** | a. Normal BMI( <25kg/m^2^ for Asians; <23 kg/m^2^ for other race )  b. Normal waist circumference(<80 cm for Asian women; <90 cm for Asian men; <88 cm for other women; <102 cm for other men)  c. Normal normoglycemia(fasting blood glucose [FBG] <100 mg/dL, glycated hemoglobin [HbA1c] <5.7%)  d. Normal normotension(systolic blood pressure [BP] <130 mmHg, diastolic BP <80 mmHg)  e. Normal lipid profile(triglycerides <135 mg/dL)  f. no evidence of chronic kidney disease(CKD) or subclinical or clinical cardiovascular disease(CVD) |
| --- | --- |
| **CKM Stage 1** | Participants without the presence of other metabolic risk factors or CKD but with any of the following:  a. Elevated BMI (≥23 kg/m² for Asians; ≥25 kg/m² for others)  b. Elevated waist circumference (≥80 cm for Asian women; ≥90 cm for Asian men; ≥88 cm for other women; ≥102 cm for other men)  c. Prediabetes (Fasting blood glucose ≥100–124 mg/dL or HbA1c between 5.7% and 6.4%) |
| **CKM Stage 2** | Participants with metabolic risk factors or moderate-to-high-risk CKD per KDIGO guidelines. Metabolic risk factors are as follows:  a. elevated fasting triglycerides (≥135 mg/dL)  b. hypertension  c. diabetes  d. metabolic syndrome (≥3 of the following: elevated waist circumference, low HDL [<40 mg/dL for men, <50 mg/dL for women], fasting triglycerides ≥150 mg/dL, elevated blood pressure [systolic ≥130 mmHg, diastolic ≥80 mmHg, or antihypertensive use], or prediabetes). |
| **CKM Stage 3** | Participants with any of the following:  a. Very-high-risk CKD (stage G4 or G5 CKD or very high risk per KDIGO classification)  b. ≥20% 10-year CVD risk(10-year cardiovascular risk estimated with the AHA Predicting Risk of CVD EVENTs (PREVENT) equations based on recommended thresholds[https://professional.heart.org/en/guidelines-and-statements/prevent-calculator]) |
| **CKM Stage 4** | Participants with self-reported established CVD, including coronary heart disease, angina, myocardial infarction, heart failure, and stroke(Atrial fibrillation and peripheral artery disease were excluded due to data unavailability). |
